# ASXL1/2 mutations and myeloid malignancies

**DOI:** 10.1186/s13045-022-01336-x

**Published:** 2022-09-06

**Authors:** Edward A. Medina, Caroline R. Delma, Feng-Chun Yang

**Affiliations:** 1 Division of Hematopathology, Department of Pathology and Laboratory Medicine, Joe R. and Teresa Lozano Long School of Medicine, University of Texas Health Science Center at San Antonio, 7703 Floyd Curl Drive, San Antonio, TX 78229-3900 USA; 2 Department of Cell Systems and Anatomy, Joe R. and Teresa Lozano Long School of Medicine, University of Texas Health San Antonio, San Antonio, TX 78229 USA; 3 Mays Cancer Center, University of Texas Health San Antonio, San Antonio, TX 78229 USA

**Keywords:** ASXL1/2, Epigenetic regulation, Hematopoiesis, Hematopoietic stem cells, His leukemogenesis, Myeloid malignancies

## Abstract

Myeloid malignancies develop through the accumulation of genetic and epigenetic alterations that dysregulate hematopoietic stem cell (HSC) self-renewal, stimulate HSC proliferation and result in differentiation defects. The polycomb group (PcG) and trithorax group (TrxG) of epigenetic regulators act antagonistically to regulate the expression of genes key to stem cell functions. The genes encoding these proteins, and the proteins that interact with them or affect their occupancy at chromatin, are frequently mutated in myeloid malignancies. PcG and TrxG proteins are regulated by Enhancers of Trithorax and Polycomb (ETP) proteins. ASXL1 and ASXL2 are ETP proteins that assemble chromatin modification complexes and transcription factors. *ASXL1* mutations frequently occur in myeloid malignancies and are associated with a poor prognosis, whereas *ASXL2* mutations frequently occur in AML with *t*(8;21)/RUNX1-RUNX1T1 and less frequently in other subtypes of myeloid malignancies. Herein, we review the role of ASXL1 and ASXL2 in normal and malignant hematopoiesis by summarizing the findings of mouse model systems and discussing their underlying molecular mechanisms.

## Introduction

Hematopoietic stem cells (HSCs) continuously replenish blood cells of the myeloid (e.g., red blood cells, granulocytes, monocytes, and platelets/megakaryocytes) and lymphoid (e.g., B and T cells) lineages through successive steps of lineage commitment. HSCs have the ability to either self-renew or differentiate to prevent their depletion in the bone marrow. Myeloid neoplasms are clonal diseases of hematopoietic stem or progenitor cells (HSC/HPCs) that are classified based on criteria applied strictly to patient-derived material acquired prior to starting therapy. These criteria are based on clinical, laboratory, hematologic, morphologic, immunophenotypic, cytogenetic, and molecular genetic features. Myeloid malignancies include the pre-leukemic states such as myeloproliferative neoplasms (MPN), myelodysplastic/myeloproliferative neoplasms (MDS/MPN), and myelodysplastic syndromes (MDS), and acute myeloid leukemia (AML) (e.g., AML with recurrent genetic abnormalities, and AML not otherwise specified) [1].

Myeloid malignancies develop through the accumulation of genetic and epigenetic alterations that dysregulate HSC function and lead to the expansion of genetically heterogeneous mutated HSCs. Clonal selection then leads to either the expansion of HSC/HPCs and hyperproliferation of particular myeloid lineages (i.e., MPN), or differentiation defects in HSCs and impaired myeloid cell production (i.e., MDS or bone marrow failure syndromes), or AML with the acquisition of driver mutations [2]. Chromosome karyotyping led to the discovery of the first gene alteration in a myeloid neoplasm, the *BCR-ABL* fusion gene, resulting from *t*(9;22)-derived Philadelphia chromosome in patients with chronic myelogenous leukemia (CML) [3]. The BCR-ABL fusion protein has constitutive tyrosine kinase activity that stimulates prooncogenic signaling in hematopoietic cells [4]. Subsequently, additional fusion genes caused by chromosomal translocations were identified, such as *RUNX1-RUNX1T1* in AML with *t*(8;21)(q22;q22), *CBFB-MYH11* in AML with inv(16)(p13.1q22) or *t*(16;16)(p13.1;q22), and *PML-RARA* in acute promyelocytic leukemia with *t*(15;17)(q22;q21). However, additional genetic alterations are required for the development of CML or AML as these fusion genes alone are not sufficient to cause leukemia [5–7]. Mechanistic studies of myeloid neoplasms, particularly AML, indicate that these disorders are hierarchically organized, stem cell (i.e., malignant/leukemic stem cell)-propagated diseases. Initiating alterations acquired within HSCs, such as the above-mentioned translocations or particularly mutations in epigenetic regulators, are early events that generate “pre-leukemic” stem cells. Pre-leukemic mutations confer HSCs with a competitive advantage without causing the transformation of downstream progenitor cells. Upon acquiring further mutations or epigenetic changes, the resultant malignant stem cells transform and lose their ability to differentiate into multiple cell lineages [8–11]. Indeed, several lines of evidence suggest that *RUNX1-RUNX1T1* is acquired in pre-leukemic HSCs, and that additional cooperating somatic mutations such as in *ASXL1* and particularly *ASXL2* are required for leukemic transformation [10, 12, 13].

Next-generation sequencing of myeloid malignancies has identified a number of recurrent, disease-causing somatic mutations. Clonal hematopoiesis of indeterminate potential (CHIP) refers to the clonal expansion of HSCs due to somatic mutations in people who do not otherwise meet the criteria for a hematologic malignancy. CHIP portends an increased risk of developing a hematologic malignancy [14] and underscores the concept that myeloid malignancies develop initially from a pre-leukemic stem cell that accumulates additional genetic alterations to become a malignant/leukemic stem cell with dysregulated self-renewal, enhanced proliferation, and impaired ability to differentiate into HPCs [15]. The genes altered in myeloid malignancies encode proteins that regulate a broad array of functions, such as signaling pathway proteins [e.g., fms-like tyrosine kinase 3 (FLT3) and Janus kinase 2 (JAK2)]; transcription factors [e.g., CCAAT enhancer-binding protein alpha (CEBPA), runt-related transcription factor 1 (RUNX1)]; tumor suppressor proteins [e.g., tumor protein p53 (TP53)]; and spliceosome components (e.g., splicing factor 3B subunit 1 (SF3B1)). Notably, a large number of somatic mutations in myeloid neoplasms are epigenetic regulators [16]. Indeed, the genes encoding the polycomb group (PcG) and trithorax group (TrxG) of epigenetic regulators, and the proteins that interact with them or affect their occupancy at chromatin, are frequently mutated in cancer, including myeloid malignancies [17, 18]. The identification of these genetic alterations in myeloid malignancies has revealed genotype–phenotype associations, enabled the characterization of their molecular function and development of targeted therapies, and facilitated stratification of a patient’s risk of disease-associated morbidity and mortality. Herein, we will review the role of ASXL1 and ASXL2 in normal and malignant hematopoiesis by summarizing the findings of mouse model systems and discussing their underlying molecular mechanisms.

**Table 1:** Mouse models used to study the functions of ASXL1/2 in myeloid malignancies

Mouse model	HSC phenotype	Disease phenotype	Disease type	Histone modification	References
*Asxl1*^−/−^	Decreased LSK	Dysplastic neutrophils, multiple lineage cytopenias	MDS-like disease, MDS/MPN	Decreased H3K27me3, H3K4me3	[71]
*Asxl1*^fl/fl^ Mx1 Cre or Vav Cre	Increased LT-HSC and LSK	Cytopenias (i.e., leukopenia and anemia), erythroid dysplasia	MDS-like disease	Decreased H3K27me3	[70]
*Asxl1*^+/−^;*Nf1*^+/−^	Increased LT-HSCs	Hepatosplenomegaly, leukocytosis, anemia, thrombocytopenia, disrupted architecture, and myeloid infiltration of spleen and liver	MDS, MDS/MPN, myeloid leukemia	Increased H3K4me3	[64]
*JAK2*^*V617F*^;*Asxl1*^+*/−*^	Increased short-term ST- HSC and MEP, and decreased MPP	Leukocytosis and thrombocytosis, megakaryocytic proliferation, increased marrow and splenic erythroid precursors, disrupted splenic architecture, and myeloid infiltration	Accelerated development of MPN, and progression to myelofibrosis and sAML	Not evaluated	[69]
*Asxl1*-MT (sublethally irradiated mice transplanted with bone marrow cells harboring an *Asxl1* mutation mimicking *ASXL1* G646WfsX12)	Not evaluated	Multilineage myelodysplasia, pancytopenia	MDS-like disease, occasional progression to AML	Decreased H3K27me3	[55]
*Asxl1*^tm/+^ + *MN1* (mimicking *ASXL1* G646WfsX12)	Promoted stem cell activities in MN1 overexpression background (i.e., increased long-term colony-forming cells)	facilitates engraftment of cells overexpressing MN1	None observed	Increased H3K27me3 with attenuated correlation between H3K27me3 and gene down-regulation	[62]
*Asxl1*^G643fs/+^ (heterozygous knock-in of *Asxl1* mutant G643WfsX12 mimicking *ASXL1*^G646fs^)	Decreased LT-HSC, LSK, MP, CMP, GMP and MEP	Leukocytosis, anemia, thrombocytosis, myeloid dysplasia in the BM, splenomegaly, disrupted splenic architecture, myeloid, and perivascular infiltration in liver	Recapitulates human low-risk MDS with some mice developing MDS/MPN-like disease after a long latency	Decreased H2AK119Ub	[72]
*Asxl1*-MT KI (Asxl1 mutant knock-in mimicking *ASXL1* E635RfsX15)	Decreased LSK, LT-HSCs and MPP, and increased MEPs	Mild leukopenia, and anemia, thrombocytosis, hypocellular bone marrow, myeloid skewing	CHIP	Decreased H3K4me3, H2AK119Ub, H3K27me3	[73, 74]
*Asxl1*^Y588X^Tg (Vav1 promoter-driven *Flag-Asxl1*^*Y588X*^ transgenic)	Increased ST-HSC and LSK, increased GMP, and decreased CMP	Hepatosplenomegaly, myeloid sarcoma, myeloid infiltration	Myeloid leukemia, MPN, MDS, MDS/MPN	Increased H3K122Ac and H3K27Ac, and alters H2AK119Ub1 occupancy at promoters of *Hoxa genes*	[75]
*Bap1*^Δ/+^; *Asxl1*^Y588X^Tg	Normalization of GMP and CMP	Normalization of spleen size	Prevented the development of myeloid malignancies	Partially restored H2AK119Ub1 occupancy at promoters of *Hoxa genes*	[76]
*Asxl2*^−/−^	Increased HSC and LSK	Pancytopenia, splenomegaly, disrupted splenic architecture, myeloid infiltration in spleen	MDS-like disease	Increased or decreased signals (> 1000 regions) for H3K27ac, H3K4me1, or H3K4me2	[77]
*Asxl2*^fl/fl^ Mx1 Cre	Decreased HSC and LSK	Granulocytic and erythroid dysplasia, leukopenia, thrombocytopenia,	MDS-like disease	Increased H3K27Ac, H3K4me1	[61]

## ASXL1 and ASXL2 protein structure

Polycomb group (PcG) and trithorax group (TrxG) genes encode essential regulators of development and differentiation in animals. PcG and TrxG proteins modulate the expression of numerous genes by regulating histone methylation to maintain repressive and active chromatin states at target loci, respectively. They preserve epigenetic memory and maintain the expression patterns of cell lineage-defining genes during cell division and replication. During embryogenesis, PcG and TrxG proteins perpetuate the spatiotemporal expression patterns of homeobox genes (*Hox* genes), which encode transcription factors that define the identity of body segments (e.g., axial skeleton, limbs, and organs) along the anterior/posterior axis in animals. PcG proteins maintain the inactive state of *Hox* genes, while TrxG proteins keep *Hox* genes activated [19]. *Hox* genes are also essential for myelopoiesis; dysregulated Hox protein expression plays a role in leukemogenesis [20].

**Fig. 1:**
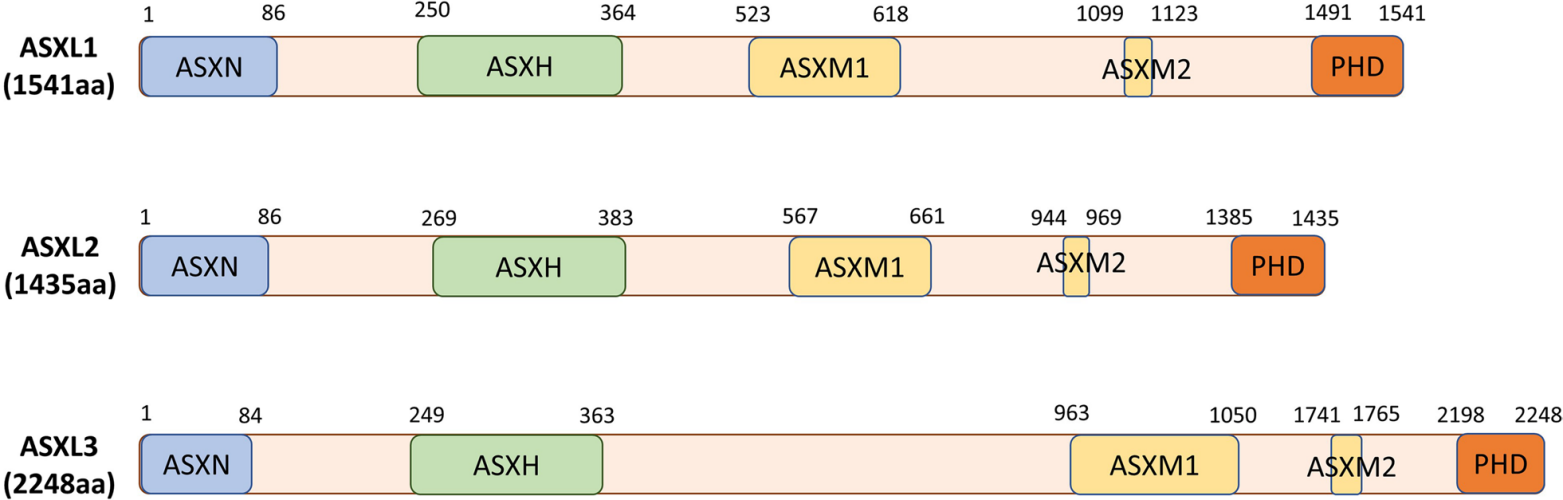
Conserved domains of the ASXL family. The N-terminal ASXN domain, ASXH and ASXM domains and the C-terminal plant homeodomain (PHD) are highly conserved in the ASXL family. The HARE-HTH motif in ASXN is a putative DNA binding domain. The ASXH domain contains a DEUBAD domain that activates BAP1 to deubiquitinate H2AK119. The LVxxLL motif in the ASXM2 domain is the binding motif for nuclear hormone receptors (NHRs) and the C-terminal PHD domain is a putative histone-recognition module

PcG and TrxG proteins are regulated by Enhancers of Trithorax and Polycomb (ETP) proteins, which recruit PcG and TrxG complexes to target chromatin. The *Drosophila* gene *Additional sex combs* (*Asx*) encodes an ETP protein. Mutations in *Asx* enhance the phenotypes of both PcG and TrxG gene mutations [21]. The mammalian homologs of *Asx* are *ASXL1-3* [22]. In an evaluation of 1004 human cell lines, *ASXL1* and *ASXL2* were found to be expressed across most of the cell lines, whereas *ASXL3* expression was more tissue specific with strong enrichment in neuroendocrine cell lines [23]. ASXL proteins do not have a catalytic domain but rather function as epigenetic scaffolds that assemble chromatin modification complexes and transcription factors. The *ASXL* genes contain 13 exons and 12 introns and share a conserved architecture with an ASXN domain at the N-terminus, an ASX homology (ASXH) domain in the N-terminal adjoining region, ASXM1 and ASXM2 domains in the middle region, and a PHD domain in the C-terminal region (Fig. 1). The ASXN domain (also known as the HB1, ASXL, restriction endonuclease helix-turn-helix, or HARE-HTH domain) is a putative DNA binding domain [24]. While the deubiquitinase adaptor domain (DEUBAD) has been described by some investigators as being in the ASXH domain [25] and others in the ASXM domains [26], there appears to be agreement that it spans approximately amino acids 238–390 of ASXL1 and 261–380 of ASXL2 [25–27], and that it is critical to binding by the deubiquitinating enzyme (DUB) BRCA-1-associated protein 1 (BAP1) [26]. The ASXM1 and ASXM2 domains additionally appear to mediate protein–protein interactions with nuclear hormone receptors (NHRs) such as PPARγ [28], and sex steroid [28, 29] and retinoic acid receptors [30]. The C-terminal PHD fingers are “readers” of histone post-translational modifications with many binding unmodified or methylated H3K4 [31] (Fig. 1).

## *ASXL1 *and *ASXL2* mutations in myeloid malignancies

### *ASXL1* and *ASXL2* mutation frequency and mutual exclusivity

Mutations in PcG and TrxG genes disrupt the specification of anterior/posterior positional information and lead to gain-of-function (posterior) and loss-of-function (anterior) homeotic transformations [32]. Both *Asxl1*^*−/−*^ and *Asxl2*^*−/−*^ mice exhibit abnormalities of axial skeletal patterning (posterior and anterior), which suggests disruption of both PcG and TrxG activities [21, 22, 33]. Thus, as regulators of PcG and TrxG activities, the ASXL proteins are crucial in maintaining the lineage-specific gene expression patterns during cell division and replication. Indeed, germ-line de novo truncating variants in human *ASXL1*, *ASXL2*, and *ASXL3* cause the congenital and developmental disorders Bohring-Opitz, Shashi-Pena, and Bainbridge-Ropers syndromes [34], respectively, and somatic mutations of *ASXL1*, *ASXL2*, and *ASXL3* occur in solid and hematologic malignancies [22, 35–37]. *ASXL1* mutations occur frequently in MDS/MPNs, particularly chronic myelomonocytic leukemia (CMML) (~ 43–49% of patients) [38, 39] and juvenile myelomonocytic leukemia (JMML) (7–8%) [40, 41], frequently in MDS (15–21%) [42, 43], and occasionally in MPN (8–10%) [44, 45], AML (3–10%) [46, 47], and CHIP (< 4%) [37]. *ASXL1* and *ASXL2* mutations occur occasionally (approximately 10%) and frequently (10–30%) in AML with *t*(8;21)/*RUNX1-RUNX1T1*, respectively [12, 48–50]. However, *ASXL2* alterations occur much less frequently in other myeloid malignancies, for example *t*(8;21)-negative de novo and secondary AML (1–3.5%) [51–53], neutrophilic leukemias of ambiguous diagnosis (CNL, aCML, MPN-unclassified, MDS/MPN, and MDS/MPN-U) (3.2%) [36], and CHIP (< 0.5%) [37].

Most *ASXL1* mutations in myeloid malignancies are frameshift or nonsense mutations in exon 12 (last exon) before the PHD finger, enabling the resulting mutant mRNA to escape nonsense-mediated decay and generate a stable C-terminal truncated ASXL1 [54, 55]. These mutations are always heterozygous, suggesting that they either have dominant-negative or gain-of-function effects. Similar *ASXL1* mutations are found in CHIP [56]. The most commonly detected *ASXL1* mutation is *ASXL1* NM_015338.5:c.1934dup;p.Gly646Trpfs*12 (ASXL1 c.1934dupG), a frameshift mutation in *ASXL1* exon 12 due to a one nucleotide expansion of a contiguous repeat of eight guanine nucleotides (GGGGGGGG) that results in a truncated ASXL1 protein lacking the PHD finger [57, 58]. *ASXL2* mutations in myeloid malignancies, at least in AML with *t*(8;21)/*RUNX1-RUNX1T1*, are out-of-frame frameshift mutations, exclusively heterozygous, and occur exclusively in exons 11 and 12 [12].

Given that ASXL1 and ASXL2 proteins have a conserved domain architecture, share a common expression pattern in embryogenesis and hematopoiesis, and are encoded from paralogous regions of the genome [DNMT3B-ASXL1-KIF3B (20q11.21)] and [DNMT3A-ASXL2-KIF3C (2p23.3)] loci, respectively, ASXL1 and ASXL2 may have overlapping or partially redundant functions. Mutations in the *ASXL* genes are frequently associated with alterations in *RUNX1*. Interestingly, *ASXL1* and *ASXL2* mutations are mutually exclusive in *t*(8;21)/*RUNX1-RUNX1T1* AML, raising the possibility that they have convergent downstream and/or synthetic lethal effects when they co-occur [59]. Indeed, mouse models have demonstrated that both *ASXL1* and *ASXL2* alterations are able to cooperate with AML1-ETO to accelerate leukemogenesis [60, 61]. However, recent evidence indicates that ASXL1 and ASXL2 have distinct, nonoverlapping functions in hematopoiesis. For example, unlike *ASXL1* alterations, *ASXL2* alterations do not co-occur with *RUNX1* point mutations, indicating that there must be differences in their leukemogenic mechanisms [12]. Additionally, Micol et al. demonstrated that *Asxl2* deletion in mice, compared with *Asxl1* deletion, led to a more pronounced leukopenia and thrombocytopenia with associated megakaryocytic hypoplasia. HSC/HPCs from mice with *Asxl2* versus *Asxl1* loss exhibited a larger number of differentially expressed genes; only a small number of dysregulated genes were shared between *Asxl2*- and *Asxl1*-deficient HSC/HPCs [61]. A mechanistic explanation for the mutual exclusivity of *ASXL1* mutations with *ASXL2* mutations in myeloid malignancies remains to be clarified.

### Cooperative leukemogenic effect of *ASXL1* alteration and co-occurring somatic alterations

**Fig. 2:**
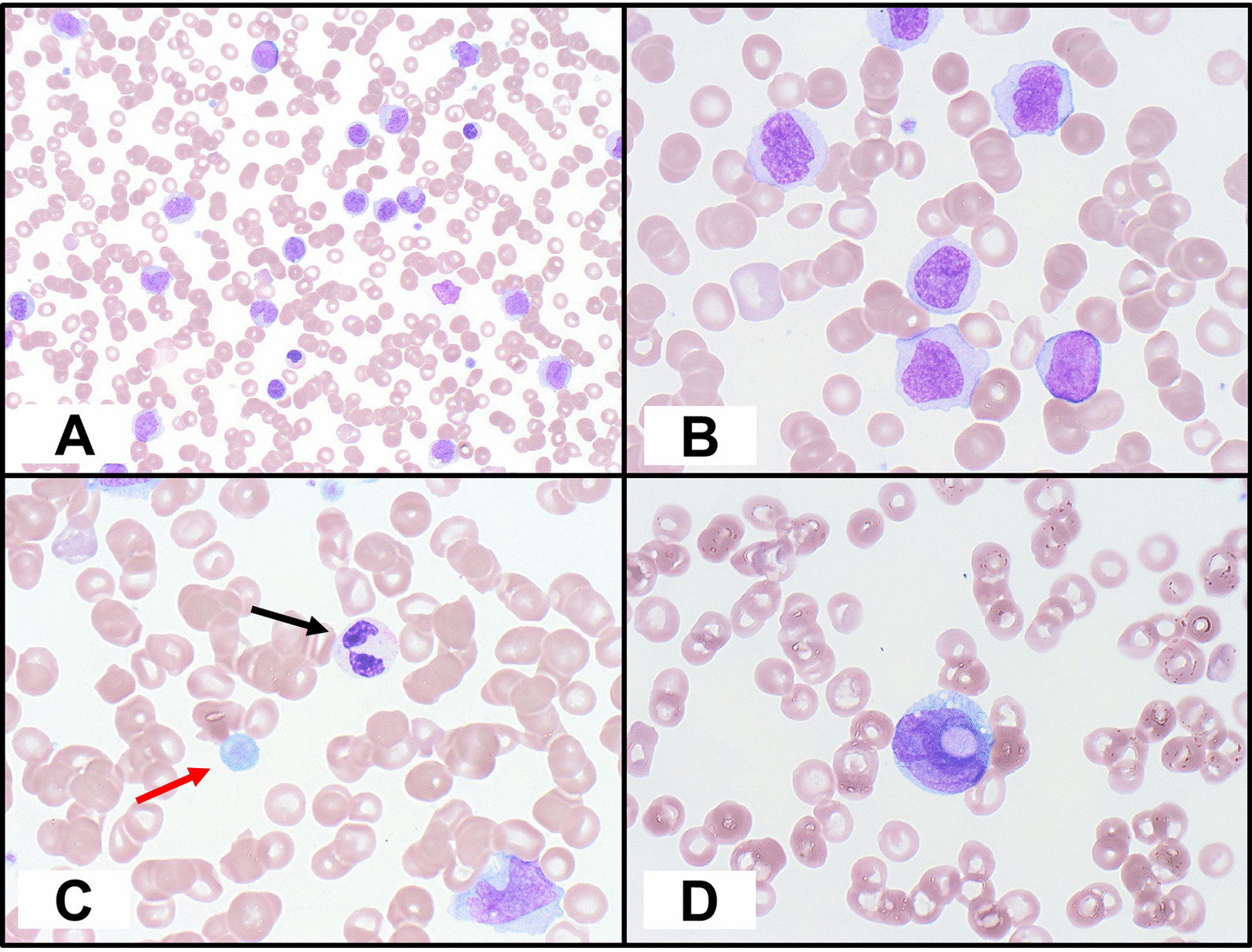
Acute myeloid leukemia with myelodysplasia-related changes (acute monocytic subtype) with interstitial del13q, *ASXL1* mutation (c.1900_p.Glu635fs), *RUNX1* mutation (c.805 + 2 T > A), and two *NRAS* mutations (NRAS c.182A > G, p.Gln61Arg and NRAS c.35G > A, p.Gly12Asp). **A** Blood smear with a myeloid blast, monoblast, several promonocytes/abnormal monocytes, and occasional dysplastic neutrophils. Higher magnification of blood smear demonstrating **B** a myeloid blast and promonocytes/abnormal monocytes, **C** dysplastic neutrophil–hyposegmented and hypogranular (black arrow), and an abnormally large and hypogranular platelet (red arrow), and **D** erythrophagocytosis by a leukemic blast following chemotherapy

As discussed, myeloid malignancies develop following the accumulation of genetic alterations in HSCs. Indeed, mutations in *ASXL1* and *ASXL2* co-occur with other gene mutations to promote leukemogenesis. Mutated *Asxl1* (mimicking *ASXL1* G646WfsX12 mutation) was shown to be able to lower the threshold for the engraftment of HSC/HPCs (from *Asxl1 *^*tm/*+^ mice) overexpressing the proto-oncogene *meningioma 1* (*MN1*) in recipient mice [62]. The neurofibromin 1 gene (*NF1*) encodes neurofibromin, a RAS GTPase activating protein that, when mutated, results in hyperactive RAS signaling that is pro-leukemogenic [63]. We and others have reported that *NF1* mutations are recurrent in AML [64, 65]. We further demonstrated that combined haploinsufficiency of *Asxl1* (*Asxl1*^+/−^) and *Nf1* (*Nf1*^+/−^) accelerates leukemogenesis in mice [64]. Moreover, *NRasG12D* induces MPN-like diseases in a bone marrow transplant model, and co-transduction of mutated *ASXL1*, but not wild-type *ASXL1*, with *NRasG12D* was shown to accelerate disease progression compared with *NRasG12D* alone [55]. Indeed, in our hematopathology service, we have observed co-occurring *ASXL1* and *NRAS* mutations in myeloid malignancies (Fig. 2). The *JAK2*V617F mutation is the most frequent genetic alteration leading to Philadelphia chromosome-negative MPNs (95% of PV and 50–60% of ET and MF). JAK2V617F  activates pro-oncogenic signaling via diverse STAT-dependent and STAT-independent pathways [66]. *JAK2* mutations are associated with age-related clonal hematopoiesis [67]. Indeed, Sidon et al. reported that *JAK2*V617F mutation was detected in 10% of blood samples from healthy volunteers [68], underscoring the necessity of additional genetic alterations for progression to an MPN. We demonstrated that *JAK2*V617F-positive PV patients harboring mutated *ASXL1* exhibited poor myelofibrosis-free survival, and that mutated *ASXL1* was present in 25% of post-PV myelofibrosis cases compared with only 4% of PV cases without myelofibrosis. We further showed that mice with heterozygous loss of *Asxl1* (*Asxl1*^+*/−*^) and JAK2V617F expression accelerated the development of bone marrow fibrosis and progression to MPN and secondary AML [69]. Interestingly, mutations in *ASXL2*, but not *ASXL1*, appear to significantly co-occur with *FLT3-ITD* mutations in AML with *t*(8;21)/*RUNX1-RUNX1T1* [12], suggesting a difference in their leukemogenic mechanisms.

## Summary of mouse models used to evaluate ASXL1 AND ASXL2 function

A number of mouse models have been developed in order to decipher the function of ASXL1 and ASXL2 in hematopoiesis and myeloid malignancies (Table 1). Homozygous or hematopoietic-specific loss of *Asxl1* results in the abnormal self-renewal capacity of HSCs and the development of MDS or MDS/MPN-like disease [70, 71], with HSC/HPCs from the latter exhibiting decreased global levels of H3K27me3 and the increased expression of posterior *Hoxa* genes [70]. We found that, compared with wild type (WT), *Asxl1*^+*/−*^, or *Nf1*^+*/−*^, compound haploinsufficiency of *Asxl1* (*Asxl1*^+*/−*^) and *Nf1* (*Nf1*^+*/−*^) in mice (*Asxl1*^+*/−*^;*Nf1*^+*/−*^) resulted in the increased frequency of long-term HSCs (LT-HSCs) in the marrow, increased genome-wide H3K4me3 occupancy, which accelerated myeloid malignancies, and development of pronounced anemia and thrombocytopenia (i.e., MDS, MDS/MPN, and myeloid leukemia) [64]. To understand the impact of altered Asxl1 and JAK2V617F expression on HSC/HPC function and MPN progression, we crossed JAK2V617F transgenic mice with *Asxl1*^+*/−*^ mice. While both *JAK2*^V617F^ with WT *Asxl1* and *JAK2*^V617F^*;Asxl1*^+/−^ mice developed MPN (i.e., PV, ET, and MF), MPN occurred sooner in the latter with more progressing to myelofibrosis and secondary AML (sAML) [69].

Mouse models of ASXL1 truncation mutations have been pivotal to understanding the mechanisms by which they contribute to leukemogenesis. Sublethally irradiated mice transplanted with bone marrow cells harboring an *Asxl1* mutation derived from the most common *ASXL1* mutation (G646WfsX12) demonstrated multilineage myeloid dysplasia, occasional progression to acute leukemia, and markedly decreased H3K27me3 levels around the promoter regions of *Hoxa* genes in the bone marrow cells from the mice developing MDS [55]. Overexpression of MN1 in HSC/HPCs from *Asxl1* mutant (mimicking *ASXL1* G646WfsX12) heterozygous knock-in mice promoted stem cell activities and lowered the threshold for engraftment in recipient mice [62]. Another heterozygous *Asxl1* mutation knock-in mouse model (*Asxl1*^G643fs/+^) recapitulated human MDS and MDS/MPN-like disease after a long latency; decreased H2AK119Ub1 deposition in HSCs due to mutant *Asxl1* resulted in the leukemogenic derepression of senescence-associated genes [72]. Conditional *Asxl1* mutant (mimicking *ASXL1* E635RfsX15) knock-in mice develop anemia, erythroid dysplasia, thrombocytosis, clonal hematopoiesis with age, and acute leukemia with the introduction of additional mutations. HSCs from these mice have an impaired function; they exhibit reductions in global H2AK119Ub and particularly H3K4me3, which lead to the decreased expression of genes involved in erythropoiesis [73, 74]. Additionally, transplantation assays using HSC/HPCs from this mouse model demonstrated that mutated *Asxl1* accelerated the development of leukemia due to AML1-ETO9a (truncated form with enhanced leukemogenicity) expression (60). We generated a mouse model expressing a C-terminal-truncated ASXL1^aa1−587^, the analogous protein product of the mutant *ASXL1*^*Y591X*^ frequently seen in patients, that developed various myeloid malignancies. Compared with WT HSC/HPCs, *Asxl1*^*Y588X*^Tg HSC/HPCs demonstrated enhanced function accompanied by increased levels of H3K122Ac and H3K27Ac, more open chromatin around transcription start sites, and dysregulated expression of genes critical for HSC self-renewal and differentiation. [75]. We discovered that truncated ASXL1 gained an interaction with BRD4, and epigenetic drug sensitivity screening demonstrated that bone marrow cells from these mice were highly sensitive to BET bromodomain inhibitors (BETis) [75]. In addition, we reported that *ASXL1* truncation mutations enhanced BAP1 deubiquitinase (DUB) activity, and that genetically reducing BAP1 in *Asxl1*^*Y588X*^Tg mice prevented the development of myeloid malignancies [76]. Constitutive homozygous loss or hematopoietic-specific deletion of *Asxl2* was shown to impair the self-renewal capacity of HSCs and lead to MDS-like disease in mice [61, 77]. Alterations in gene expression due to *Asxl2* loss were associated with dysregulated H3K27ac and H3K4me1/2 [77]. Hematopoietic-specific deletion heterozygous loss of *Asxl2* was further demonstrated to cooperate with AML1-ETO to promote leukemogenesis in mice as evidenced by their decreased survival, increased circulating blasts, and extramedullary leukemic infiltration [61].

## The molecular mechanisms underlying ASXL1/2-mediated HSC/HPC functions

### Regulation of gene expression by histone modifications

Post-translational modifications of histones, such as acetylation, methylation, and ubiquitination, modify chromatin compaction and stability. Lysine acetylation of histones (e.g., H3K9, H3K9, H3K18, and H3K27) is associated with open chromatin and active transcription [78, 79]. Histone methylation can cause gene activation (H3K4, H3K36, and H3K79) or repression (H3K9, H3K27, and H4K20) [79]. Ubiquitinated H2AK119 is associated with gene silencing via its role in regulating the deposition of methylated H3K27 [80]. PcG proteins form large multimeric complexes involved in gene silencing through modifications of chromatin organization; they silence hundreds of developmental decision-makers and signaling factors. They have been subdivided into the polycomb repressive complex (PRC) 1 and PRC2 groups. Canonical PRC1 (cPRC1) compacts polynucleosomes independently of its histone ubiquitinase activity, variant PRC1 (vPRC1) ubiquitinates H2AK119 (H2AK119Ub1), and PRC2 methylates H3K27 (H3K27me1/2/3) [81]. How PRCs are targeted to silence genomic targets is unclear [82, 83]. TrxG proteins form multiprotein complexes with H3K4 methyltransferase activity and counteract PcG-mediated gene silencing by depositing H3K4me2/3 [84].

### ASXL1/2 and PRC1 complex

**Fig. 3:**
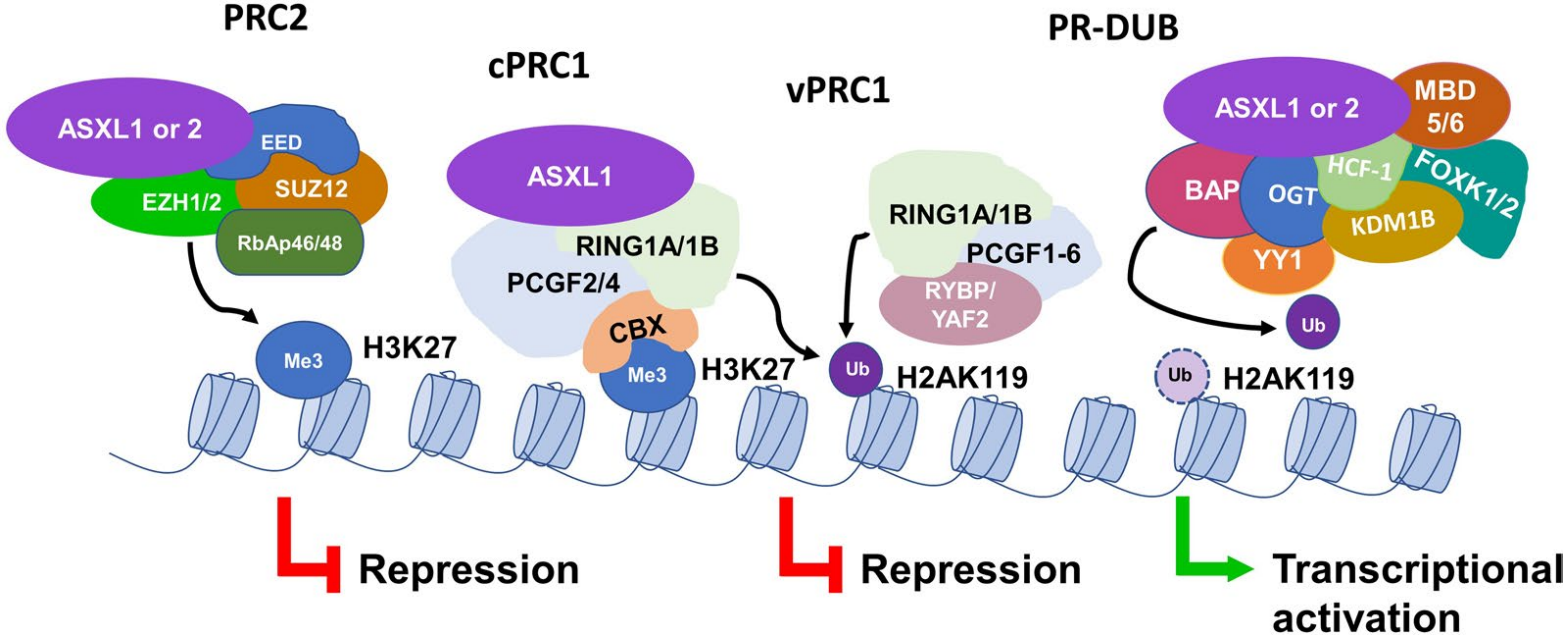
ASXL1/2 and multiprotein complex regulation of gene expression by histone modifications. ASXL1 and ASXL2 each associates with the histone methyl transferase EZH1/2, SUZ12, EED and RbAp46/48 to form a complex (i.e., PRC2) that recognizes H2AK119Ub1, and methylates H3K27 (H3K27me1/2/3) leading to the silencing of target genes. PRC1 complexes contain an E3 ubiquitin ligase that monoubiquitinates H2AK119 (H2AK119Ub1). The CBX protein of canonical PRC1 (cPRC1) binds H3K27me3, which enables H2AK119Ub1 deposition, and additionally interacts with nucleosomes for chromatin compaction and transcriptional repression. The variant PRC1 (vPRC1) recognizes H2AK119Ub1 via RYBP or YAF2 to propagate H2AK119Ub1. PR-DUB complex, comprised of ASXL1 or ASXL2, the deubiquitinase BAP1, and various interacting proteins, deubiquitinates H2AK119Ub1 resulting in the derepression of target genes. Loss of *Asxl1* in HSC/HPCs has been shown to cause a global loss of H3K27me3 and the increased expression of leukemogenic genes. *ASXL1* truncation mutations result in a hyperactive mutation-ASXL1/BAP1 complex that depletes H2AK119Ub1 resulting in a loss of H3K27me3 and increased expression of leukemogenic genes. ASXL2 loss in HSC/HPCs results in a slight reduction of H3K27me3 levels

The activities of ubiquitin-activating (E1), conjugating (E2), and ligase (E3) proteins result in the attachment of one or several ubiquitin molecules (i.e., mono- or polyubiquitination, respectively). Conversely, ubiquitin is removed by deubiquitinase enzymes [85]. To date, eight different PRC1 complexes have been identified. Each contains an E3 ubiquitin ligase, a really interesting new gene (RING) 1A or 1B, and a distinct set of subunits that influence PRC1 function. The various PRC1 complexes are classified as cPRC1 if they have a chromodomain (CBX) protein and either polycomb group RING finger protein 4 (PCGF4) (aka BMI1) or PCGF2 (MEL18), and vPRC1 if they have one of the six PCGF factors and one of the two homologous proteins RING1 and YY1 binding protein (RYBP) or YY1-associated factor 2 (YAF2) [82, 86, 87]. RYBP and YAF2 are readers of H2AK119Ub1 that recruit vPRC1 to propagate H2AK119Ub1 on neighboring nucleosomes [88]. The DNA-binding activities of vPRC1 complexes also influence their recruitment to target sites. For example, the vPRC1 complex PRC1.1 interacts with KDM2B, an H3K36me2 demethylase that binds to nonmethylated CpG islands through its zinc finger-CxxC DNA-binding domain, which recruits the complex to silence particular polycomb target genes [89]. Thus, the variation in subunit composition of the individual PRC1 complexes enables localization to distinct genomic loci and the fine-tuning of gene repression in response to diverse stimuli. While PRC1 catalysis is essential for PcG-mediated gene silencing, the mechanism by which H2AK119Ub1 mediates repression is unclear. The current evidence suggests that cPRC1, via its CBX protein, binds H3K27me3 deposited by PRC2 and then deposits H2AK119Ub1. cPRC1 additionally compacts chromatin to repress target genes through interactions between CBX protein and nucleosomes. vPRC1 exhibits strong catalytic activity and is recruited independently of PRC2 and H3K27me3 to catalyze H2AK119Ub1, which then targets PRC2 to chromatin for the deposition of H3K27me3 at PcG targets [87, 90, 91] (Fig. 3). Notably, the removal of vPRC1 complexes caused a near-complete loss of H2AK119Ub1 and widespread reactivation of PcG target genes [91]. Indeed, loss of H2AK119Ub1 induces a rapid displacement of PRC2 activity and loss of H3K27me3 deposition, thereby underscoring the role it plays in PcG-mediated repression [80].

Modeling the most commonly detected *ASXL1* mutation using *Asxl1*^G643fs/+^ mice (heterozygous knock-in of *Asxl1* mutant G643WfsX12 mimicking *ASXL1*^G646fs^) demonstrated abnormal hematopoiesis and recapitulated human low-risk MDS with some mice developing MDS/MPN-like disease after a long latency. These pro-leukemogenic effects appeared to be caused by impaired PRC1 activity in HSCs due to the loss of interaction between the ASXL1 mutant and BMI1, which led to the derepression of senescence-associated genes (e.g., *p16Ink4a*) as a result of decreased H2AK119Ub1 deposition around their promoter regions [72]. The importance of the ASXL1-BMI axis to H2AK119Ub1 and neutrophil differentiation was corroborated in zebrafish with endogenous heterozygous Asxl1 C-terminal mutation. *Asxl1* mutation resulted in decreased *bmi1* and *cbx4* expression, decreased global H2AK119Ub1 levels, impaired neutrophil differentiation, neutrophilic dysplasia, and in some mutants, monocytosis or increased myeloblasts [92]. On the other hand, as discussed further below, ASXL1 truncations have been shown to impart a gain-of-function enhancement of the histone H2A deubiquitinase activity of the ASXL1–BAP1 complex [93]. Thus, multiple mechanisms may be mediating the decline in H2AK119Ub1 levels that occurs with *ASXL1* mutations. To our knowledge, no interactions between ASXL2 and PRC1 core components have been reported.

### ASXL1/2 and PRC2 complex

Both ASXL1 [94] and ASXL2 [95] have been shown to interact with the core subunits of PRC2. The PRC2 complex methylates H3K27 and is comprised of four core subunits: the histone methyltransferase Enhancer of Zeste Homolog 2 (EZH2) or EZH1, Suppressor of Zeste 12 (SUZ12), Embryonic Ectoderm Development (EED), and Retinoblastoma protein-associated protein 46/48 (RbAp46/48) (Fig. 3). The core PRC2 subunits associate with equimolar stoichiometries, and all are required for the catalytic activity of the complex [96, 97]. In addition to the core components, there are a number of associated molecules, including AE binding protein 2 (AEBP2), Jumonji and AT-rich interaction domain containing 2 (JARID2), PHD finger protein 19 (PHF19), polycomb-like proteins (PCLs), and the long intergenic noncoding RNA HOTAIR [83]. There are two subtypes of PRC2: PRC2.1 [containing a PCL homolog (PCL1-3) along with EPOP (C17ORF96) or PALI (C10ORF12)], and PRC2.2 (containing JARID2 and AEBP2). These interacting molecules play crucial roles in regulating complex recruitment to targets and enzymatic activity [97]. For example, JARID2 links PRC1 and PRC2 to establish repression at target gene loci by mediating the interaction of PRC2 with H2AK119Ub1 [98].

Abdel-Wahab et al*.* demonstrated that ASXL1 associates with EZH2, and that loss of *ASXL1* in an AML cell line (UKE1) and human cord blood CD34 + cells causes global loss of H3K27me3 as well as the increased expression of posterior *HOXA* cluster genes (i.e., *HOXA5-9*). The reintroduction of WT ASXL1 protein restored H3K27me3 levels and suppressed *HOXA* gene expression in leukemia cells with mutated *ASXL1*. However, neither an interaction between ASXL1 and the PRC1 component BMI1 nor changes in H2AK119Ub1 levels with ASXL1 loss were evident in the AML cell lines evaluated (SET2 and/or UKE1 cells) [94]. These investigators subsequently demonstrated that HSC/HPCs from *Asxl1* deficient mice exhibited decreased global levels of H3K27me3 and the increased expression of posterior *Hoxa* genes [70]. Sublethally irradiated mice (Ly5.1) transplanted with bone marrow cells (from Ly5.2 mice) harboring a C-terminal-truncating *Asxl1* mutation (derived from the mutated genes of 1934dupG;G646WfsX12 and 1900–1922del;E635RfsX15 of patients with MDS) exhibited multilineage myeloid dysplasia and occasional progression to acute leukemia. ChIP analysis of the promoter regions of posterior *Hoxa* genes using H3K27me3 antibodies found that H3K27me3 was markedly decreased around the promoter regions of *Hoxa5*, *Hoxa9*, and *Hoxa10* in the bone marrow cells of mice with MDS [55]. Overall, these findings do establish the critical role that ASXL1 plays in regulating PRC2 activity at PcG target genes and support the notion that C-terminally-truncated  ASXL1promotes leukemogenesis via the loss of PRC2-mediated gene repression.

ASXL2 has been shown to interact with the core PRC2 subunits EZH2 and SUZ12 to enrich target promoters with repressive H3K27me3 in mouse heart tissue [95], and H3K27me3 levels are markedly reduced in the hearts of *Asxl2*^*−/−*^ mice [33]. In contrast, we and others have recently reported a slight reduction of H3K27me3 levels in HSC/HPCs or bone marrow mononuclear cells from Asxl2^−/−^ mice compared with those from Asxl1^−/−^ mice [61, 77]. Notably, Micol et al. further demonstrated that while ASXL1 interacted with the core PRC2 subunit SUZ12 in 293 T cells, ASXL2 did not [61]. It is possible that the differences in the effects of *Asxl2* loss on H3K27me3 levels between cardiac and hematopoietic cells, at least in mice, are tissue/cell-type-specific. Thus, the role that ASXL2 plays in regulating PRC2 complexes in hematopoiesis requires further study.

### Polycomb repressive deubiquitinase (PR-DUB) complexes

#### Role of ASXL1/2 in PR-DUB complexes

H2AK119Ub1 is catalyzed by PRC1 and reversed by BRCA1-associated protein 1 (BAP1) deubiquitinase complexes [23] (Fig. 3). Calypso and additional sex combs (Asx) form the polycomb repressive deubiquitinase (PR-DUB) complex in *Drosophila*. Calypso is the catalytic DUB within the complex, which hydrolyzes H2AUb when activated by Asx [86]. BAP1 is the mammalian homolog of Calypso; it is a major DUB of H2AK119Ub1 [99].

BAP1 is stabilized and activated by ASXL1–3; ASXL1/ASXL2 deletion in cells leads to the complete degradation of BAP1 protein without affecting BAP1 transcript levels [100]this sentence  and the four sentences that follow below should all be a part of paragraph one. The ubiquitin C-terminal hydrolase (UCH) imparts the DUB activities of the PR-DUB in Calypso and BAP1, while the C-terminal domain (CTD) interacts with DEUBAD of Asx or ASXL proteins; a stable interaction between CTD and DEUBAD is required for BAP1 DUB activity [27, 82, 86, 99]. The CTD of BAP1 interacts with monoubiquinated DEBAUD domain, which allosterically increases BAP1’s affinity for ubiquitin on H2A at Lys-119 H2A and stimulates deubiquitination [27, 99]. BAP1 promotes monoubiquitination of DEBAUD by recruiting ubiquitin ligases; its DUB activity stabilizes ASXL2 and ASXL1 by preventing ubiquitin chain elongation and subsequent proteasomal degradation [26, 101]. Thus, monoubiquitination of DEBAUD regulates the DUB activity of BAP1 and stabilizes the ASXL proteins. *ASXL1* mutations give rise to truncated proteins that retain the amino-terminal, BAP1-interacting region of ASXL1 [26, 54]. Importantly, BAP1 was shown to have a higher affinity for C-terminally truncated ASXL1 (*ASXL1* p.E635RfsX15) compared with WT ASXL1, leading to the dramatic stabilization of mutated ASXL1, increased stability of BAP1, and enhanced monoubiquinated DEBAUD and BAP1 DUB activity [101]. A hyperactive truncated ASXL1/BAP1 complex was further demonstrated to result in the global reduction of H2AK119Ub1 and H3K27me3, upregulation of *HOXA* and interferon regulatory factor 8 (*IRF8*) expression, and impairment of mouse HSC/HPC function [101]. These findings are corroborated by Balasubramani et al. who further demonstrated that truncated ASXL1 (*ASXL1* p.G646WfsX12)-BAP1 complexes in a multipotent hematopoietic precursor cell line (EML cells) confer gain-of-function on BAP1 DUB activity that leads to the marked global reduction of H2AK119Ub1 and H3K27me3, upregulation of mast cell- and basophil-specific genes whose promoters are marked by these repressive histone modifications, and skewed differentiation to the mast cell lineage [93]. Additionally, Wang et al. showed that CRISPR-Cas9-generated frame shift mutations producing truncated ASXL1 in THP1 cells resulted in BAP1 stabilization and its enhanced localization to the promoter region of several pro-leukemogenic genes found to be upregulated [102]. We recently demonstrated that mice expressing C-terminal-truncated ASXL1^aa1−587^ (pan-hematopoietic *Vav1* promoter-driven *Asxl1*^Y588X^ transgenic mice) developed various myeloid malignancies (i.e., AML, MPN, MDS, and MDS/MPN) [75]. We found that truncated ASXL1 competed with full-length ASXL1 for binding to BAP1, and reduced H2AK119Ub1 occupancy at the promoters of *Hoxa* and *Dcbld1* genes in HSC/HPCs, resulting in their increased expression. Notably, deleting one *Bap1* allele in these mice (i.e., *Bap1*^Δ/+^; *Asxl1*^Y588X^Tg) partially restored H2AK119Ub1 occupancy at the promoters of these genes, normalized their expression, and prevented the development of myeloid malignancies [76]. Thus, *ASXL1* truncation mutations confer gain-of-function in the pathogenesis of myeloid malignancies by increasing BAP1 DUB activity, and reducing BAP1 activity ameliorates the abnormal hematopoietic phenotypes due to mutated *Asxl1*. Because most *ASXL2* mutations are out-of-frame frameshift mutations in exons 11 and 12, at least in AML with *t*(18;21) [12], the BAP1 binding region in ASXL2 is also unaffected, although it remains to be determined whether truncated ASXL2-BAP1 complexes exhibit enhanced DUB activity. ASXL1 and ASXL2 compete for interaction with BAP1; BAP1-ASXL1 and BAP1-ASXL2 complexes have been shown to be present at similar levels in cells. Cancer-associated *BAP1* mutations that inhibit the interaction between ASXL1/2 and BAP1 and impair DUB activity result in deregulated cell proliferation. Cancer-associated *BAP1* mutations also result in impaired cellular senescence. BAP1-ASXL2, but not ASXL1-BAP1 complexes, appears to mediate this tumor-suppressive function; overexpression of BAP1 or ASXL2, but not ASXL1, induces senescence, and deletion of the ASXM domain of ASXL2 impairs senescence (human fibroblasts IMR90 cell line) [99]. While hematopoietic-restricted BAP1 loss in mice has been reported to lead to myeloproliferative [103] or MDS-like disease [104], somatic *BAP1* mutations, unlike in some solid tumors (e.g., malignant mesothelioma and melanoma), are rarely present in myeloid malignancies [105]. Indeed, Dey et al. reported that out of 32 patients with de novo MDS, a somatic *BAP1* mutation was detected in one case [104]. Nevertheless, it remains to be explored whether the impairment of BAP1 interactions with ASXL1/2 due to *BAP1* mutation leads to deregulation of cell proliferation and/or inhibition of cellular senescence in myeloid malignancies. The tumor-suppressive function of the ASXL1-BAP1 axis was further highlighted by Cao et al. These investigators demonstrated that *Asxl1* loss enabled IL-3 independent growth in an AML cell line (murine 32D cells) due to the increased activation of AKT resulting from the increased levels of H2AK119Ub1 (globally as well) and H3K27me3 at the *Pten* promoter, which maintained *Pten* silencing (PTEN negatively regulates the PI3K-AKT pathway). They further demonstrated that overexpression of WT ASXL1 , but not ASXL1 with deletion of amino acids 1–400 at the N-terminus (results in loss of BAP1 binding), restored PTEN expression in *Asxl1*^−/−^ cells. These data confirm that ASXL1 -induced PTEN expression depends onBAP1 DUB activity, and links deregulation of ASXL1 to dysregulation of the PTEN/AKT signaling axis in myeloid malignancies [106].

#### BAP1-interacting proteins

BAP1 associates tightly with ASXL proteins to form a core complex and forms larger complexes as a result of transient interactions [100] with transcription factors [i.e., Yin Yang 1 (YY1) [107], forkhead transcription factors FOXK1/2 [104, 108], chromatin binders and modifiers [i.e., the lysine-specific demethylase KDM1B, O-linked N-acetylglucosamine transferase (OGT)] [104], the cell cycle regulator: host cell factor 1 (HCF-1) [107] and DNA repair proteins methyl-CpG-binding domain family (MBD5/6) [109] (Fig. 3). BAP1 also deubiquitinates and stabilizes some of its interacting proteins, such as HCF1 and OGT; BAP1 loss in hematopoietic cells decreases OGT and HCF-1 levels [104]. Thus, in addition to chromatin modification, BAP1’s interactions with these proteins enable the regulation of diverse cellular functions such as proliferation, differentiation, metabolism, cell death, and DNA damage repair [110, 111].

Interestingly, Xia et al*.* recently reported that ASXL1 interacted with forkhead transcription factors FOXK1 and FOXK2 to upregulate FOXK1/K2 target genes, such as the tumor suppressors Von Hippel-Lindau syndrome (*VHL*), thioredoxin interacting protein (*TXNIP*), and suppressor of cytokine signaling 1 and 2 (*SOCS1*, *SOCS2*), that regulate oxygen sensing, glucose metabolism/redox homeostasis, and JAK-STAT signaling, respectively. They further evaluated the impact of deleting endogenous mutant *ASXL1* (C-terminal-truncated) in a background of additional oncogenic alterations using leukemia cell lines (i.e., K562 and Kasumi cells). Deletion of mutant *ASXL1* allele significantly decreased cell cycle progression/proliferation, increased serum starvation-induced apoptosis, and increased the expression of FOXK1/2 target genes. They demonstrated that while mutated *ASXL1* enhanced BAP1 activity and led to decreases in global H2AK119Ub1, H2AK119Ub1 levels were increased at specific genomic loci (i.e., FOXK1/2 target genes). *ASXL1* mutants were shown to impair the association of wild-type ASXL1 with BAP1 in a dominant-negative manner by sequestering the enzyme and suppressing its recruitment to the promoters of FOXK1/K2 target genes, thereby leading to the maintenance of H2AK119Ub1 levels; deleting mutated *ASXL1* restored the expression of BAP1-ASXL1-FOXK1/K2 target genes that play a role in hematopoiesis and/or tumor suppression (e.g., VHL, SOCS1/2, and TXNIP). Overall, the findings from this study raise the possibility that impairment of BAP1-ASXL1-FOXK1/K2 axis due to mutated *ASXL1* is leukemogenic [112]. ASXL2-BAP1 interactions have been shown to play a role in the suppression of solid tumorigenesis (e.g., human mesotheliomas and lung cancers), and ASXL2-BAP1 complexes, similar to ASXL1-BAP1 complexes, demonstrated to interact with BAP1-interacting proteins (e.g., YY1, FOXK1/2, OGT, and HCF1) [99]. However, while the role of ASXL2-BAP1 complexes and their associated proteins in normal and malignant hematopoiesis can be inferred from studies with solid malignancies, their precise function remains to be established.

### ASXL1/2 and TrxG histone methylation activities

#### COMPASS-like complexes

**Fig. 4:**
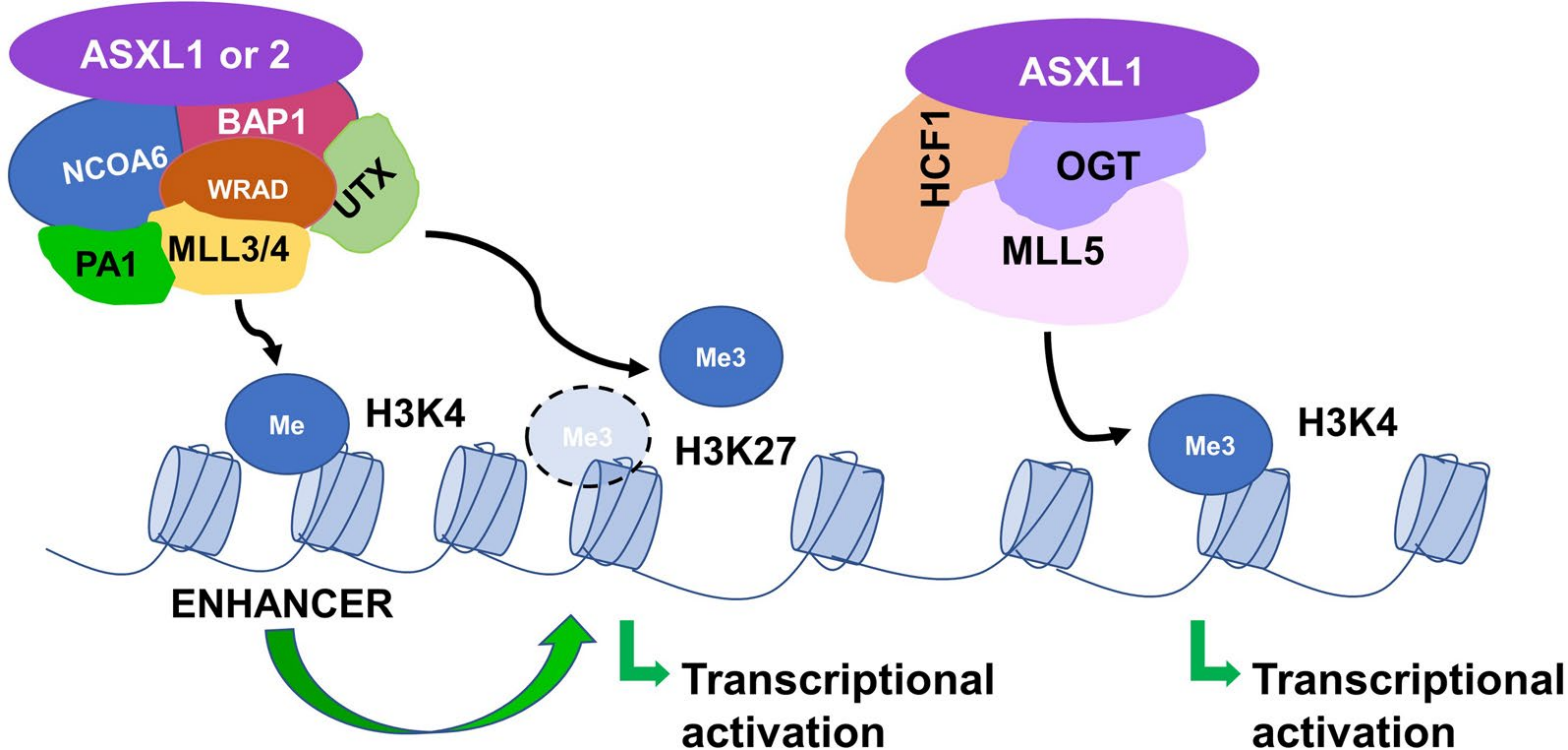
ASXL1/2 and trithorax gene activation by MLL3/MLL4 COMPASS and MLL5. MLL3/MLL4 COMPASS is composed of the WRAD complex of proteins (WDR5, ASH2, RBBP5, and DPY30), which is required for H3K4 methyltransferase activity, as well as NCOA6, PA1, BAP1 (interacts with ASXL1 or ASXL2), and the H3K27 demethylase UTX. ASXL1 interacts with OGT1 and HCFC1 to form a complex that collaborates with MLL5 for the trimethylation of H3K4. The deposition of H3K4me1 by MLL3/MLL4 at enhancers, demethylation of H3K27 by UTX, and the deposition of H3K4me3 by MLL5 facilitate transcriptional activation

TrxG proteins form complexes that maintain the activated state of target genes by methylating H3K4 (i.e., H3K4me1/2/3) [84]. Chromatin remodeling proteins such as chromodomain-helicase-DNA binding protein 1 (CHD1) recognize H3K4me2/3 and alter nucleosome positioning to facilitate DNA transcription and replication [113]. The first mammalian homolog of *Drosophila* *trx* identified was the Mixed Lineage Leukemia (*MLL*) gene, which was cloned based on its identification in recurring chromosomal translocations involving 11q23 in AML and acute lymphoblastic leukemia [114]. The function of MLL was unraveled through genetic and biochemical studies of its yeast homolog Set1. The SET (Suppressor of variegation 3–9 (Su(var)3–9); Enhancer of Zeste (E(z)); and Trithorax) domain is a lysine methyltransferase (KMTase), capable of methylating lysine residues on histones [115]. Set1 is a KMTase that is active only as part of a complex named Complex of Proteins Associated with Set1 (COMPASS). The yeast Set1/COMPASS is the founding member of the family of COMPASS H3K4 methyltransferases, which catalyze H3K4me1/2/3. In mammals, six Set1-related enzymes (SET1/MLL family members)—Set1A, Set1B, and MLL1-4 (excludes MLL5)—reside in COMPASS-like complexes with the WRAD complex of proteins [WD repeat-containing protein 5 (WDR5), Absent-Small-Homeotic-2- Like protein (ASH2), Retinoblastoma Binding Protein 5 (RBBP5), and Dumpy-30 (DPY30)], which is required for methyltransferase activity, as well as additional proteins. For example, MLL3/MLL4 COMPASS also includes Nuclear Receptor Coactivator 6 (NCOA6), PA1, and the H3K27me3 demethylase Ubiquitously Transcribed Tetratricopeptide Repeat on chromosome X (UTX) [116, 117] (Fig. 4). Mammalian Set1A/Set1B is responsible for genome-wide H3K4me3 deposition, MLL1/MLL2 catalyzes H3K4me3 at specific loci, and MLL3/MLL4 deposits H3K4me1 at enhancers [84, 118, 119] (Fig. 4).

#### MLL3/MLL4 compass

The overall evidence to date suggests that BAP1 is not involved in the repression of PcG target genes, but rather antagonizes PRC2-mediated silencing by (1) opposing PRC1-mediated H2A ubiquitination [23, 100] (Fig. 3); and (2) recruiting MLL3/MLL4 and the H3K27me3 demethylase (UTX) at enhancers to deposit H3K4me1 and demethylate H3K27me3, respectively [86, 117] (Fig. 4). The balance in the activity between PRC1, PRC2, BAP1, and COMPASS-like complexes determines whether a gene is active or silenced. We demonstrated that loss of *Asxl1* leads to MDS-like disease in mice. HSC/HPCs from *Asxl1*^−/−^ mice exhibited increased apoptosis and mitosis, similar to human MDS, and the increased expression of *Hoxa* genes (*Hoxa5/7/9/10*), abnormal expression of genes regulating apoptosis (e.g., *Bcl2* downregulated), as well as reduced global levels of H3K27me3 and H3K4me3 [71]. To mimic the common human *ASXL1* mutation, p.E635RfsX15, conditional C-terminal truncated *Asxl1* mutant knock-in (*Asxl1*-MT KI) mice were generated that impaired hematopoiesis (e.g., myeloid skewing, age-dependent mild anemia with erythroid differentiation block, modest dysplasia, and thrombocytosis) but was not sufficient to transform HSCs. However, when combined with additional mutations provided by insertional mutagenesis (MOL4070LTR retrovirus) or expression of mutant *RUNX1* (Runt-related transcription factor 1), mutant *ASXL1* expression increased the susceptibility of HSCs to transform. Notably, *Asxl1*-MT KI mice displayed substantial reductions in global H3K4me3 and H2AK119Ub1, and focal reductions in H3K27me3 [73]. Thus, *ASXL1* alteration itself or concomitant mutations decrease PRC1, PRC2, and COMPASS-like activity at PcG target genes, leading to leukemogenesis.

#### MLL5

MLL5 contains a SET domain (typically exhibits KMTase activity) and was previously considered to be a member of the SET1/MLL family; however, it differs from the rest of the family in that it lacks intrinsic KMTase activity toward histones [120]. Multiple studies indicate that MLL5 plays a critical role in normal and malignant hematopoiesis [121]. MLL5 was shown to regulate cell cycle progression by interacting with HCF-1 and OGT, which rendered it capable of depositing H3K4me3 at E2F1 target promoters to activate transcription [122]. Supporting a role for MML5 in mediating trithorax functions of ASXL1, Inoue et al*.* demonstrated that ASXL1 interacts with OGT1 and HCFC1 to form a complex that collaborates with MLL5 to trimethylate H3K4 (Fig. 4) and regulate myelopoiesis [123]. Modification by GlcNAcylation can influence a protein in a variety of ways, such as modulating its enzyme activity, stability, subcellular localization, or interacting proteins [124]. OGT was found to stabilize ASXL1 via GlcNAcylation, and knockdown of ASXL1, OGT, HCF1, or MLL5 blocked ATRA-induced myeloid differentiation and decreased H3K4 methylation, altering the expression of genes involved in myeloid differentiation, ribosomes/translation, and mRNA splicing/spliceosomes in HL-60 cells (AML cell line) [123]. Thus, an impaired ASXL1–MLL5 axis may explain the decrease in H3K4me3 levels that occurs in HSC/HPCs with *ASXL1* loss or from mutated *Asxl1*-MT KI mice [73]. Nevertheless, an evaluation of the involvement of other members of the SET1/MLL family in mediating ASXL trithorax functions is required. It remains to be determined whether ASXL2 has similar interacting partners as ASXL1. We demonstrated that *Asxl2 *loss dysregulated H3K27ac and H3K4me1/2 and altered the expression of genes critical for HSC self-renewal, differentiation, and apoptosis in HSC/HPCs [77]. Micol et al. corroborated these findings demonstrating by ChIP-seq analysis that *ASXL2* loss (following RNAi-mediated *ASXL2* depletion in the myeloid leukemia cell line SKNO-1) was associated with increases in signals for H3K27ac and/or H3K4me1 at enhancers (active/poised) [61].

#### ASXL1 and cohesin complex

Cohesin is a ring-shaped protein complex that mediates sister chromatid cohesion, DNA replication, DNA repair and dynamic restructuring of chromosomes during cell division, and gene regulation. Cohesin is composed of three core subunits (SMC1A, SMC3, and RAD21), and additional regulatory proteins (i.e., STAG1, STAG2, PDS5A, PDS5B, NIPBL, WAPL, and Sororin) that modulate its activity. Cohesin regulates gene expression by mediating the extrusion of chromatin to form a DNA loop structure; extrusion halts when it encounters the 11-zinc-finger transcription factor CTCF. When a cohesin-mediated DNA loop forms or is lost between a promoter and an enhancer, gene expression is either increased or decreased, respectively. Moreover, enhancer activity is limited to the region inside of the cohesin-mediated DNA loop (anchored by pairs of distal CTCF sites) [125]. While cohesin has been demonstrated to be a putative tumor suppressor in hematologic malignancies, leukemias with cohesin mutations are not associated with chromosomal instability (e.g., aneuploidy). Rather, cohesin mutations confer a clonal advantage by altering the chromatin and transcriptional state of HSC/HPCs and perturbing the balance between self-renewal and differentiation [126, 127].

We demonstrated that ASXL1 interacts with the three core proteins (SMC1A, SMC3, and RAD21) of cohesion. Deletion of *Asxl1* resulted in a significantly higher frequency of hematopoietic cells, demonstrating nuclear bridging and impaired telophase chromatid disjunction. Disrupting cohesin and endogenous ASXL1 interaction by expressing ASXL1 amino acids 401 to 587 (i.e., the cohesin-binding region of ASXL1) increased the frequency of cells with nuclear bridging and premature sister chromatid separation. Moreover, ChIP-seq analysis of HSC/HPCs from WT and Asxl1^−/−^ mice demonstrated significant overlap of ASXL1/SMC1A/RAD21 binding sites (93% of genomic binding sites at promoter regions); *Asxl1* loss resulted in the decreased genomic occupancy of cohesin, and the dysregulation of several ASXL1/SMC1A/RAD21 common target genes, including those that have a role in apoptosis, proliferation, and myelopoiesis and/or leukemogenesis (e.g., *Stat3*, *Cbfb*, and *Fus*). Taken together, these findings indicate that ASXL1 interaction with cohesin is required to maintain normal cell morphology, chromatid separation, and gene expression in hematopoietic cells [128].

## Therapeutic targeting of mutant ASXL1 in myeloid malignancies

**Fig. 5:**
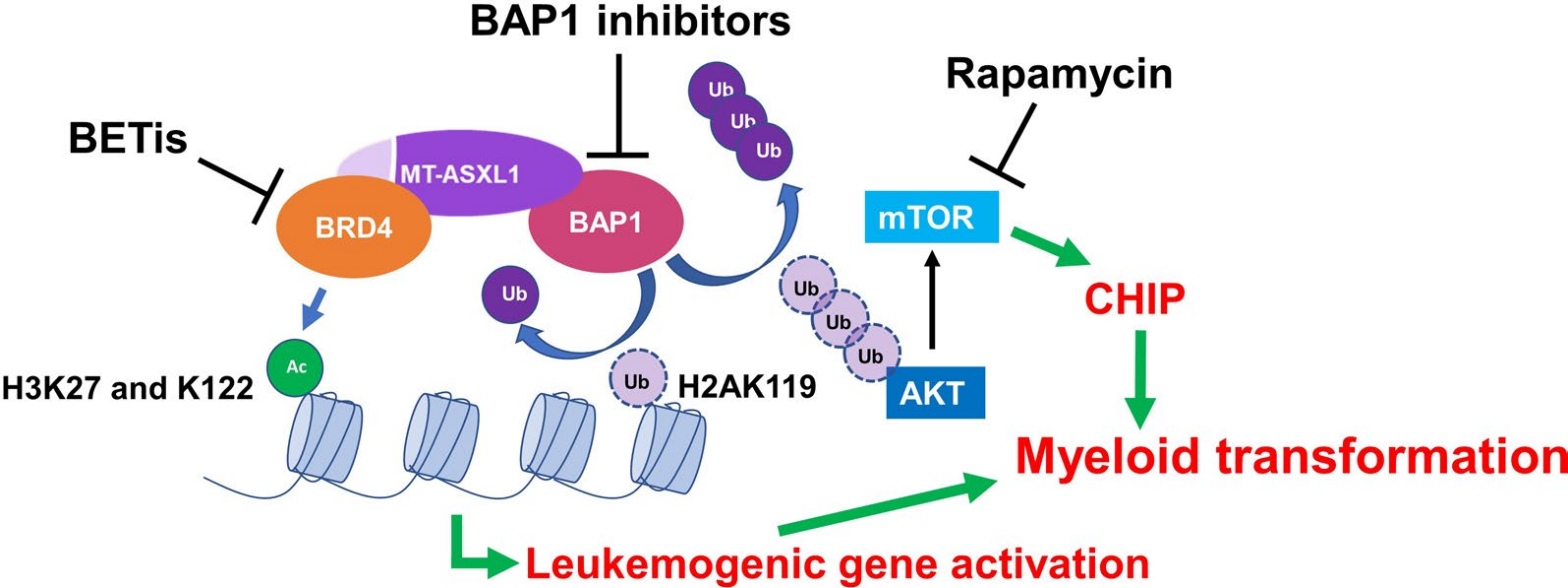
Therapeutic targeting of mutant ASXL1 in myeloid malignancies. Mutated ASXL1 (MT-ASXL1) stabilizes BAP1, resulting in a hyperactive MT-ASXL1/BAP1 complex that demonstrates increased localization to leukemogenic loci and subsequent reduction of repressive H2AK119Ub1 and H3K27me3. Targeting BAP1 decreases MT-ASXL1/BAP1 complex leukemogenicity. MT-ASXL1/BAP1 complex also deubiquinates and stabilizes AKT, resulting in enhanced signaling through AKT/mTOR pathway, leading to HSC/HPC dysfunction, clonal hematopoiesis of indeterminate potential (CHIP), and increased risk of leukemogenesis. Pharmacologic inhibition of mTOR (i.e., rapamycin) abrogates abnormal hematopoiesis. MT-ASXL1 gains interaction with bromodomain-containing protein 4 (BRD4) leading to aberrant acetylation of H3 (e.g., H3K27 and H3K122Ac), marks associated with open chromatin and active transcription, leading to leukemogenic gene activation and HSC/HPC dysfunction. BET bromodomain inhibitors (BETis) reduce the levels of H3K122Ac and H3K27Ac and normalize HSC/HPC functions

Fujino et al. recently reported that while *Asxl1* mutant HSCs exhibited impaired function, these HSCs acquired a clonal advantage during aging, similar to CHIP in humans. They demonstrated that mutated ASXL1-BAP1 complex deubiquinated and stabilized AKT, which enhanced signaling through the AKT/mTOR pathway and induced aberrant cell cycle progression and proliferation of HSC/HPCs as well as abnormal mitochondrial activation, overproduction of ROS, and increased DNA damage. Notably, pharmacologic inhibition of mTOR (i.e., rapamycin) was able to abrogate the abnormal hematopoiesis (Fig. 5) in the *Asxl1*-MT KI mice [74]. The potential of targeting BAP1 in myeloid malignancies with *ASXL1* mutations (Fig. 5) was demonstrated by Wang et al. who corroborated that truncated ASXL1 stabilized BAP1 and increased its recruitment to leukemogenic gene loci for their activation and then used a biochemical screen to identify a BAP1 catalytic inhibitor that delayed disease progression in NSGS mice engrafted with leukemic cells from a patient diagnosed with de novo AML harboring an *ASXL1* truncation mutation (p.Q588*) [102].

We recently discovered that, in contrast to WT full-length ASXL1, truncated ASXL1^aa1−587^ gained interaction with bromodomain-containing protein 4 (BRD4). BRD4, a member of the bromodomain and extra terminal domain (BET) family (also includes BRD, BRD3, and BRDT), is an epigenetic reader that contains two bromodomains that recognize acetylated lysine residues. BRD4 functions as a scaffold for transcription factors at promoters and super-enhancers, and as a histone acetyltransferase (HAT) that acetylates H3K4, H3K9, H3K18, H3K27, and H3K122, marks associated with open chromatin and active transcription [78]. The dynamic balance between lysine acetyltransferase (KAT) and histone deacetylase (HDAC) enzymes determines the degree of lysine acetylation of N-terminal histone tails, a major epigenetic mark for open chromatin and active transcription. Notably, we found that bone marrow cells from *Asxl1*^Y588X^ transgenic mice were highly sensitive to BET bromodomain inhibitors (BETis), but not HDAC inhibitors, in epigenetic drug sensitivity screening. Furthermore, BETis dramatically reduced the levels of H3K122Ac and H3K27Ac in 32D cells expressing ASXL1^aa1−587^ and normalized HSC/HPC functions. These findings support a role for the ASXL1^aa1−587^–BRD4 axis in the hematopoietic phenotypes exhibited by *Asxl1*^*Y588X*^ transgenic mice and raise the possibility of targeting BRD4 in myeloid malignancies with mutated *ASXL1* (Fig. 5) [75]. Importantly, underscoring the potential of BET bromodomain inhibition, Binder et al. recently demonstrated that leukemogenic gene activation (e.g., mitotic kinases, *HOXA* cluster genes, MAPKand receptor tyrosine kinase signaling) in samples from CMML patients with truncating *ASXL1* mutations were associated with permissive promoter chromatin states due to H3K27ac and H34me1 deposition, and *ASXL1* mutation-specific de novo accessibility of distal enhancers binding ETS transcription factors, as well as BRD4 [129].

## Open questions

Our review raises a number of questions regarding the molecular mechanisms by which ASXL1 and ASXL2, and their mutated forms, exert their effects in normal and malignant hematopoiesis, respectively. For example, both ASXL1 and ASXL2 have been shown to compete for interaction with BAP1 [99]. Truncated ASXL1 has a higher affinity for BAP1 compared with WT ASXL1 [101]. Does truncated ASXL1 impact ASXL2-BAP1 complex formation and function? How might this contribute to dysregulating hematopoiesis and/or promoting leukemogenesis? Does truncated ASXL2, like truncated ASXL1, have a higher affinity for BAP1 compared with WT ASXL2 that results in a hyperactive truncated ASXL2-BAP1 complex with enhanced histone H2A DUB activity? Does truncated ASXL2 gain interaction with BRD4 that promotes leukemogenesis? While *ASXL1* alterations in HSC/HPCs lead to global loss of H3K27me3 and the aberrant expression of leukemogenic genes, *ASXL2* alterations appear to have a minimal impact on the global levels of H3K27me3 in HSC/HPCs [77]. What are the epigenetic silencing mechanisms used by ASXL2 to regulate hematopoiesis? What is the impact of the most common *ASXL2* mutations on histone modifications that regulate gene expression? The ASXL1–MLL5 axis regulates H3K4me3 levels in HSC/HPCs. What other members of the SET1/MLL family are involved in mediating ASXL1 trithorax functions? While mouse models have demonstrated that both *ASXL1* and *ASXL2* alterations cooperate with AML1-ETO to accelerate leukemogenesis, what are the underlying mechanisms? Finally, what is the mechanistic explanation for the mutual exclusivity of *ASXL1* mutations with *ASXL2* mutations in myeloid malignancies, and why are *ASXL1* mutations more prevalent in myeloid malignancies compared with *ASXL2* mutations?

## Conclusion

Studies exploring the effects of ASXL1 or ASXL2 loss demonstrated their critical role in hematopoiesis and tumor suppression via their regulation of PcG and TrxG complex activities, which antagonistically regulate the expression of genes involved in hematopoietic stem cell (HSC) self-renewal, proliferation, and differentiation into hematopoietic progenitor cells (HPCs). They have also revealed that ASXL1 interaction with cohesin is required to maintain normal cell morphology, chromatid separation, and gene expression in hematopoietic cells. The most commonly observed *ASXL1* mutations in myeloid malignancies are heterozygous and give rise to truncated ASXL1. Mouse studies have demonstrated that heterozygous *Asxl1* mutations mimicking the most common *ASXL1* mutation give rise to CHIP in older mice, and cooperating genetic alterations accelerate leukemogenesis. Efforts to elucidate the leukemogenic mechanisms of mutated *ASXL1* have revealed potential new therapeutic targets. Truncated ASXL1-BAP1 complexes confer gain-of-function on H2AK119Ub1-DUB activity that leads to decreases in H3K27me3 levels and the activation of normally silenced genes, as well as deubiquitination of AKT, which enhances signaling through the AKT/mTOR pathway to stimulate the proliferation of HSC/HPCs. Truncated ASXL1 also aberrantly associates with BRD4 and leads to dysregulated hematopoiesis. Indeed, the mTOR inhibitor rapamycin or BET bromodomain inhibitors were able to counteract some of the leukemogenic effects of mutated *ASXL1*. Finally, as the landscape of epigenetic aberrations and resulting dysregulated genes due to mutated *ASXL1* and *ASXL2* continue to be characterized, it is expected that therapeutic targeting of epigenetic regulators to restore the epigenome and transcriptome of HSC/HPCs will increasingly emerge as options to prevent progression of CHIP to the pre-leukemic myeloid malignancies or acute leukemia.

## Data Availability

Not applicable.

## Abbreviations

aCML: Atypical chronic myelogenous leukemia
AEBP2: AE binding protein 2
AML: Acute myeloid leukemia
ASH2: Absent-Small-Homeotic-2- Like protein
Asx: Additional sex combs
ASXH: ASX homology
BAP1: BRCA-1-associated protein 1
BET: Bromodomain and extra terminal domain
BETis: BET bromodomain inhibitors
BRD4: Bromodomain-containing protein 4
CBX: Chromodomain
CEBPA: CCAAT enhancer-binding protein alpha
CHD1: Chromodomain-helicase-DNA binding protein 1
CHIP: Clonal hematopoiesis of indeterminate potential
CML: Chronic myelogenous leukemia
CMML: Chronic myelomonocytic leukemia
CMP: Common myeloid progenitor
CNL: Chronic neutrophilic leukemia
COMPASS: Complex of proteins associated with set1
DEUBAD: Deubiquitinase adaptor domain
DUB: Deubiquitinase
DPY30: Dumpy-30
EED: Embryonic ectoderm development
ET: Essential thrombocythemia
ETP: Enhancers of Trithorax and Polycomb
FLT3: Fms-like tyrosine kinase 3
GMP: Granulocyte–macrophage progenitor
HARE-HTH: HB1, ASXL, restriction endonuclease helix-turn-helix
HAT: Histone acetyltransferase
HCF-1: Host cell factor 1
HDAC: Histone deacetylase
HSC: Hematopoietic stem cell
HSC/HPC: Hematopoietic stem or progenitor cell
JAK2: Janus kinase 2
JARID2: Jumonji and AT-rich interaction domain containing 2
JMML: Juvenile myelomonocytic leukemia
KAT: Lysine acetyltransferase
KMTase: Lysine methyltransferase
LSK: Lin-Sca1 + c-Kit +
LT-HSC: Long-term HSC
MBD: Methyl-CpG-binding domain
MDS: Myelodysplastic syndromes
MDS/MPN: Myelodysplastic/myeloproliferative neoplasms
MEP: Megakaryocyte-erythrocyte progenitor
MF: Myelofibrosis
MLL: Mixed lineage leukemia
MMP: Multipotent progenitor
MP: Myeloid progenitor
MPN: Myeloproliferative neoplasms
MPP: Multipotential progenitor
MN1: Meningioma 1
NCOA6: Nuclear receptor coactivator 6
NF1: Neurofibromin 1 gene
NHR: Nuclear hormone receptor
OGT: O-linked N-acetylglucosamine transferase
PCL: Polycomb-like protein
PcG: Polycomb group
PCGF4: Polycomb group RING finger protein 4
PHF19: PHD finger protein 19
PRC: Polycomb repressive complex
PR-DUB: Polycomb repressive deubiquitinase
PV: Polycythemia vera
RBBP5: Retinoblastoma binding protein 5
RbAp46/48: Retinoblastoma protein-associated protein 46/48
RING: Really interesting new gene
RUNX1: Runt-related transcription factor 1
RYBP: RING1 and YY1 binding protein
sAML: Secondary AML
SET: Suppressor of variegation 3–9 (Su(var)3–9); Enhancer of Zeste (E(z)); and Trithorax
SF3B1: Splicing factor 3B subunit 1
SOCS: Suppressor of cytokine signaling
ST- HSC: Short-term HSC
SUZ12: Suppressor of Zeste 12
TrxG: Trithorax group
TP53: Tumor protein p53
TXNIP: Thioredoxin-interacting protein
UCH: Ubiquitin C-terminal hydrolase
ULD: UCH L5-like domain
UTX: Ubiquitously transcribed tetratricopeptide repeat on chromosome X
VHL: Von Hippel-Lindau syndrome
WDR5: WD repeat containing protein 5
WT: Wild type
YAF2: YY1-associated factor 2
